# Myeloablative Temozolomide Enhances CD8^+^ T-Cell Responses to Vaccine and Is Required for Efficacy against Brain Tumors in Mice

**DOI:** 10.1371/journal.pone.0059082

**Published:** 2013-03-18

**Authors:** Luis A. Sanchez-Perez, Bryan D. Choi, Gary E. Archer, Xiuyu Cui, Catherine Flores, Laura A. Johnson, Robert J. Schmittling, David Snyder, James E. Herndon, Darell D. Bigner, Duane A. Mitchell, John H. Sampson

**Affiliations:** 1 Duke Brain Tumor Immunotherapy Program, Division of Neurosurgery, Department of Surgery, Duke University Medical Center, Durham, North Carolina, United States of America; 2 The Preston Robert Tisch Brain Tumor Center at Duke, Duke University Medical Center, Durham, North Carolina, United States of America; 3 Department of Pathology, Duke University Medical Center, Durham, North Carolina, United States of America; 4 Translational Research Program, Abramson Family Cancer Research Institute, Perelman School of Medicine, University of Pennsylvania, Philadelphia, Pennsylvania, United States of America; 5 Department of Radiation Oncology, Duke University Medical Center, Durham, North Carolina, United States of America; 6 Department of Immunology, Duke University Medical Center, Durham, North Carolina, United States of America; 7 Department of Biostatistics and Bioinformatics, Duke University Medical Center, Durham, North Carolina, United States of America; The University of Chicago, United States of America

## Abstract

Temozolomide (TMZ) is an alkylating agent shown to prolong survival in patients with high grade glioma and is routinely used to treat melanoma brain metastases. A prominent side effect of TMZ is induction of profound lymphopenia, which some suggest may be incompatible with immunotherapy. Conversely, it has been proposed that recovery from chemotherapy-induced lymphopenia may actually be exploited to potentiate T-cell responses. Here, we report the first demonstration of TMZ as an immune host-conditioning regimen in an experimental model of brain tumor and examine its impact on antitumor efficacy of a well-characterized peptide vaccine. Our results show that high-dose, myeloablative (MA) TMZ resulted in markedly reduced CD4^+^, CD8^+^ T-cell and CD4^+^Foxp3^+^ T_Reg_ counts. Adoptive transfer of naïve CD8^+^ T cells and vaccination in this setting led to an approximately 70-fold expansion of antigen-specific CD8^+^ T cells over controls. *Ex vivo* analysis of effector functions revealed significantly enhanced levels of pro-inflammatory cytokine secretion from mice receiving MA TMZ when compared to those treated with a lower lymphodepletive, non-myeloablative (NMA) dose. Importantly, MA TMZ, but not NMA TMZ was uniquely associated with an elevation of endogenous IL-2 serum levels, which we also show was required for optimal T-cell expansion. Accordingly, in a murine model of established intracerebral tumor, vaccination-induced immunity in the setting of MA TMZ–but not lymphodepletive, NMA TMZ–led to significantly prolonged survival. Overall, these results may be used to leverage the side-effects of a clinically-approved chemotherapy and should be considered in future study design of immune-based treatments for brain tumors.

## Introduction

Temozolomide (TMZ) is an alkylating agent that has demonstrated clinical benefits in patients with glioblastoma (GBM) [1] and advanced metastatic melanoma [2]; however, despite aggressive multimodal therapy, patients with these diseases suffer a dismal prognosis of less than 15 months and 8 months from the time of diagnosis, respectively [1], [2]. Spurred by the need for safer, more effective treatments, immunotherapy has emerged as a rapidly developing area of research given its capacity to eliminate cancer cells potently without toxicity [3]–[5]. Despite this great promise, translation of immune-based cancer treatment for patients with primary and metastatic brain tumors has been limited to date, partly due to challenges associated with mounting and sustaining antigen-specific T cells *in vivo*, and doing so despite the severely lymphotoxic side effects of standard-of-care TMZ [6]–[8].

A recent advance, however, is the discovery that lymphodepletion can counter-intuitively augment antitumor immunity by potentiating antigen-specific T-cell responses, specifically during the recovery phase from lymphopenia [9]–[12]. This phenomenon has not only been observed for vaccine-induced immunity [13]–[17] but also in the context of adoptive T-cell therapy where lymphodepletive host-conditioning prior to adoptive transfer has been shown to induce homeostatic proliferation, resulting in enhanced persistence, function and anti-tumor efficacy of transferred cells in both murine models and early clinical trials [18]–[22]. Indeed, recent evidence suggests that greater degrees of lymphopenia may result in even more potent immune responses [10], [18], [23], [24]; in clinical trials for patients with stage III/IV melanoma, intensive myeloablation followed by adoptive T-cell therapy led to unprecedented overall response rates of approximately 70%. A considerable drawback to such myeloablative host-conditioning regimens, however, is the need for high-dose interleukin-2 (IL-2) and treatment with chemotherapy or radiation therapy that is not only highly toxic but also fails to provide direct antitumor benefit. In contrast, dose escalation of clinically efficacious cytotoxic agents such as TMZ may conveniently provide synergistic conditioning for effective immunotherapy, without the need for additional lymphodepletive agents.

Previous reports have shown that TMZ-induced lymphopenia can serve as a means to augment antitumor T-cell responses [25]. However, despite the fact that TMZ is used routinely in patients with brain tumors [26]–[29], such effects have yet to be demonstrated appropriately in experimental models of intracerebral tumor. Moreover, little is known about serum profiles of immunopotentiating cytokines (*e.g.*, IL-2, IL-7 and IL-15) that may be elaborated upon TMZ-induced lymphodepletion and whether these levels lead to an enhancement of immune responses against tumors in the CNS [30].

In this study, we found that vaccination in the setting of myeloablative (MA) TMZ–but not lower, lymphodepletive doses–is necessary to achieve prolonged survival in a stringent mouse model of established intracerebral tumor. Data showed that MA TMZ leads to a dramatic reduction of all T-cell lymphocyte subsets, including regulatory T cells (T_Regs_). Interestingly, MA, but not lower doses of TMZ, resulted in sustained elevation of endogenous IL-2 serum levels, the presence of which was actually required for optimal T-cell expansion. Adoptive transfer and antigen-specific peptide vaccination following treatment with MA TMZ resulted in a more than 70-fold expansion of antigen-specific CD8^+^ T-cells as well as enhanced IFNγ secretion compared to controls. When administered prior to vaccine, MA TMZ host-conditioning proved superior in its capacity to prolong overall survival in mice with intracerebral tumors compared to lower-dose TMZ.

Together, our findings highlight the specific importance of MA TMZ in potentiating functional antitumor T-cell responses against tumors in the brain and have the potential to impact the design of future clinical studies for patients with brain tumors and other CNS malignancies.

## Materials and Methods

### Mice and Tumor Cell Lines

C57BL/6J and OT-I transgenic mice [31] were obtained from Jackson Laboratories. Mice were maintained and bred under pathogen-free conditions at Duke University Medical Center (DUMC). All animal experiments were performed according to protocols approved by the Duke University Institutional Animal Care and Use Committee (IACUC). The IACUC at Duke University specifically approved the study (Protocol Number: A356-09-12). The tumor cell lines B16F10 and B16F10-OVA were a kind gift from Dr. Richard G. Vile, PhD, at Mayo Clinic [32], [33].

### TMZ Preparation

TMZ (Temodar®, Schering-Plough^©^ and Best Pharmatech^©^) was dissolved in a solution of 85% saline and 15% dimethyl sulfoxide (DMSO). Mice were weighed and injected intraperitoneally with a calculated dose daily for 5 days as indicated.

### In vivo IL-2 Blockade

S4B6 and JES6-1 antibodies (BioXcell^©^) were combined and administered intraperitoneally at 250 µg per mouse. Antibody treatment was initiated the day after TMZ termination, immediately before adoptive lymphocyte transfer (ALT) and vaccine administration. Treatment was repeated every other day for a total of 7 times.

### Peripheral Blood Draws and Complete Blood Counts

Retro-orbital eye bleeding was used to collect 50–100 µl blood into heparinized tubes for complete blood count (CBC) and flow cytometric analyses. CBCs were performed by the Duke Veterinary Diagnostic Laboratory on a VetScan HM5 Hematology Analyzer (Abaxis Inc).

### Hematopoietic Stem Cell Rescue

Hematopoietic stem cell rescues (HSCs) were extracted from the bone marrow by negative selection utilizing a lineage cell depletion kit as instructed by the manufacturer (Cat# 130-090-858, Miltenyi Biotech^©^). The day after cessation of myeloablative TMZ regimen, 5 × 10^4^ cells were administered intravenously.

### Antibodies and Flow Cytometry

Antibodies to CD3 (145-2C11), CD4 (L3T4), CD8 (53-6.7), and CD16/32 (2.4G2), as well as appropriate isotype controls, were obtained from BD Pharmingen (San Diego, CA). Anti-Foxp3 (FJK-16s) was obtained from eBioscience (San Diego, CA). Whole blood obtained by retro-orbital bleeding was analyzed for T-cells, CD8^+^ T-cells, and CD4^+^ T-cells as follows: samples were incubated with a cocktail of antibodies CD3-PE, CD4-FITC and CD8-APC in 150 µl FACS buffer in the dark for 15 minutes at room temperature. Red blood cells (RBCs) were lysed and cells were fixed using 1 ml 1X BD FACS Lysing Solution (BD Bioscience, Cat# 349202) and incubated overnight at 4°C. Cells were then washed and re-suspended in 2% paraformaldehyde and submitted to flow cytometry analysis. For CD4^+^Foxp3^+^ T_Reg_ analyses, peripheral blood samples were incubated with 1X RBC lysing buffer (BD Pharm Lyse Cat# 555899) 3 times. Cells were incubated with 10 µg/ml Fc block CD16/CD32 antibodies (BD Biosciences Cat# 553142), followed by surface staining with CD4 antibody at 4°C for 30 minutes. Cells were then washed and submitted to a mouse Foxp3 intracellular staining protocol as described by the manufacturer using the anti-mouse/rat foxp3 staining set (eBiosciences Cat# 77–5775). For PE-H-2 Kb OVA (SIINFEKL murine tetramer, Beckman Coulter^©^ Cat# T03000) staining of circulating OT-I CD8^+^ T-cells, 50 µl whole blood, obtained by retro-orbital bleeding, was incubated with CD8-APC and OVA tetramer in 150 µl FACS buffer in the dark for 15 minutes at room temperature. RBCs were lysed and cells were fixed in 1 step with 1X BD FACS Lysing Solution as described above. All samples were analyzed on a FACSCalibur flow cytometer (BD Biosciences). Absolute numbers per microliter of blood were calculated using Flowcount^®^ beads from Beckman Coulter^©^ according to manufacturer instructions.

### Cytometric Bead Array

To analyze memory recall responses, 50 µl peripheral blood was collected from mice and transferred to a 96-well plate. RBCs were lysed with 1X lysing buffer (BD Pharm Lyse, #555899) and cells were washed twice and stimulated with 1 µM SIINFEKL chicken OVAlbumin peptide or negative control peptide in complete T-cell media (RPMI 10% FBS 100 µM nonessential amino acids, 1 mM sodium pyruvate, 2 mM L-glutamine, 50 µg/ml gentamycin sulfate, 100 IU/ml penicillin/Streptomycin and 55 µM 2-mercaptoethanol). Cells were incubated at 37°C with 5% CO_2_, and supernatant was collected after 72 hours. A 50 µl culture supernatant was mixed with 50 µl capture beads and 50 µl detection reagent (Mouse CBA Th1/Th2/Th17 cytokine Kit, BD Biosciences #560485) and incubated for 2 hours at room temperature. Cells were washed and analyzed by flow cytometry all per manufacturer instructions.

### MILLIPLEX Cytokine Panel

Peripheral blood was collected from mice on days 5 and 12 after the cessation of TMZ treatment. Plasma was derived and IL-2 cytokine levels were determined (MILLIPLEX MAP mouse cytokine/chemokine kit protocol, Millipore custom made kit, Cat# MPXMCYTO-70K-03) following manufacturer instructions.

### OT-I Transfer and Vaccination

Mice undergoing vehicle (Veh) or TMZ treatment received splenocytes from CD8^+^ OVA-specific TCR transgenic mice (OT-I) and normal C57BL/6 donor mice mixed at a 1∶1 ratio (1×10^7^ cells total) 1 day after cessation of TMZ. Immediately following OT-I infusion, mice were vaccinated intradermally with 100 µg OVA class I peptide (SIINFEKL; American Peptide Company, Inc., CA) in 10% DMSO with an equal volume of Complete Freund’s Adjuvant (CFA) (DIFCO Laboratories, MI) (100 µl/mouse).

### Tumor Implantation

B16F10 and B16F10-OVA cells were grown in Dulbecco’s Modified Eagle Medium (DMEM), 10% FBS and 2 mM L-glutamine at 37°C in 5% CO_2_ incubators. B16F10-OVA cells were kept under G418 antibiotic selection 2.5 mg/ml to ensure chicken OVAlbumin expression. For intracranial tumor implantation, cells were harvested, counted and mixed 50/50 with 10% methylcellulose in phosphate buffer saline, and loaded into a 250 µl syringe (Hamilton, Reno, NV) with an attached 25-gauge needle. The needle was positioned 2 mm to the right of bregma and 4 mm below the surface of the skull at the coronal suture using a stereotactic frame (Kopf Instruments, Tujunga, CA). Cells were implanted at 5.0×10^3^ in a total volume of 5 µl into C57BL/6 mice. Tumor bearing mice were monitored daily for morbidity endpoints and survival according to the Duke University IACUC guidelines.

### Statistical Analysis

Unless otherwise stated, Mann-Whitney non-parametric test was used to determine statistical significance. Survival data from the animal studies were analyzed by the Gehan-Breslow-Wilcoxon Test. Statistical significance was determined at the level of *P*<0.05. All analyses were conducted using GraphPad Prism version 5.01.

## Results

### Myeloablative TMZ Leads to Sustained Lymphopenia

**Figure 1:**
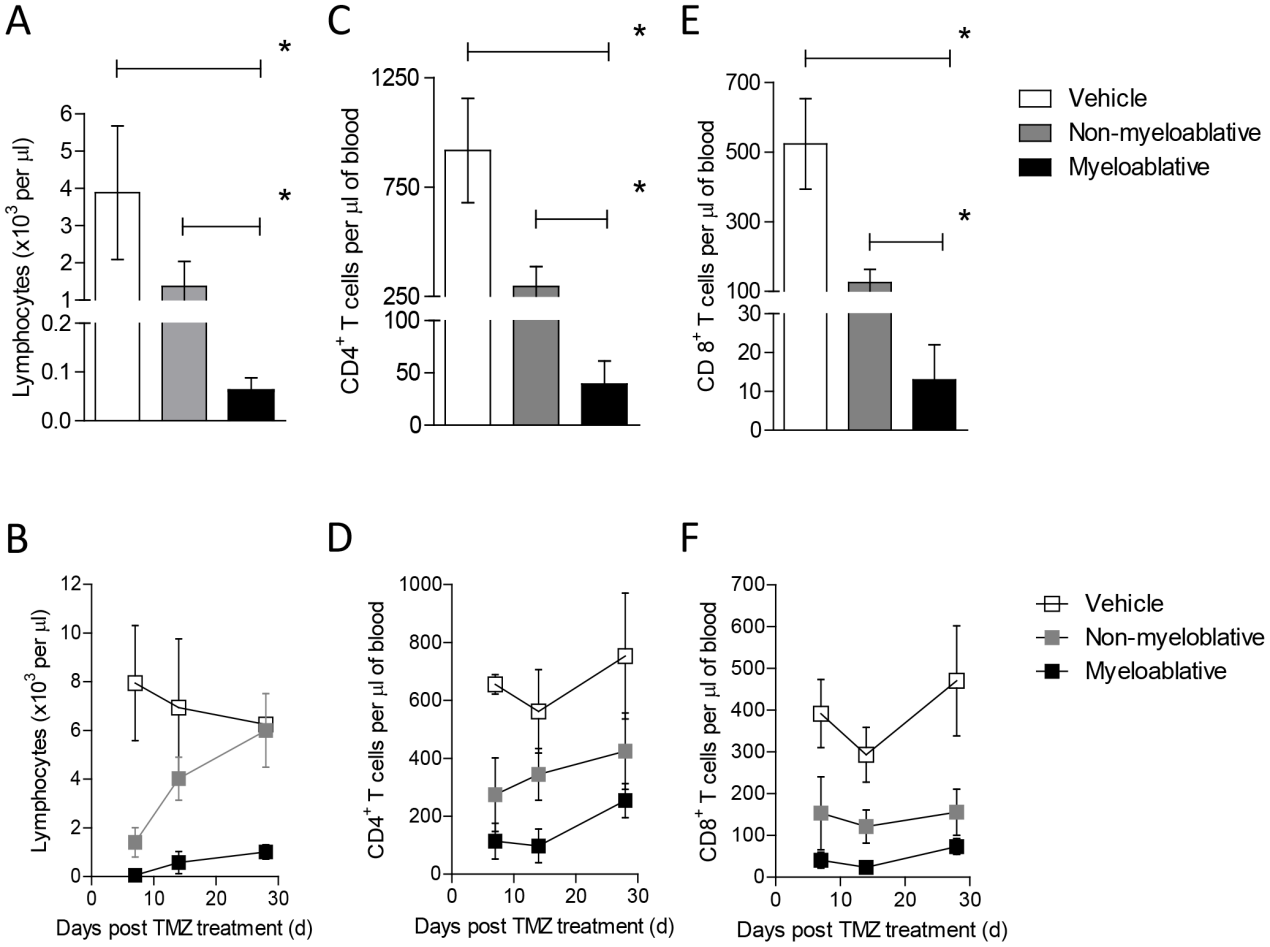
Myeloablative TMZ leads to profound and sustained CD4^+^ and CD8^+^ T-cell lymphopenia. To evaluate the degree of lymphopenia T-cell depletion induced by different TMZ regimens, wild-type C57BL/6 mice (n = 5) were treated with NMA (60 mg/kg) or MA (125 mg/kg) TMZ intraperitoneally for 5 consecutive days. Immediate effects of TMZ on lymphocyte counts (A), CD4^+^ T-cell (C) and CD8^+^ T-cell (E) on day 2 and their respective kinetics of recovery over time is shown (B,D, F ). Blood from mice bled retro-orbitally on days 2, 7, 14 and 28 after termination of TMZ, was submitted to CBC analysis for lymphocyte counts on the indicated days. FACS analyses of for CD4^+^ and CD8^+^ T-cell was performed. All experiments were performed in triplicate. Statistical analysis was performed using Mann-Whitney test. Statistical significance was determined at a *p value ≤0.05.

We established a MA TMZ regimen consisting of 125 mg/kg×5 days +50,000 Lin^-^ HSC rescue. Doses lower than 125 mg/kg×5 days were not MA, while higher doses were lethal despite HSC rescue. In order to assess the impact of MA TMZ on host immunity, peripheral blood from treated mice was subjected to CBC and flow cytometric analysis. Treatment with MA TMZ resulted in severe lymphopenia (Fig. 1A, MA vs. Veh, p = 0.0079), that was sustained over a 28-day period (Fig. 1B). The degree of lymphodepletion achieved by MA TMZ was greater than that achieved by a previously published NMA TMZ regimen of 60 mg/kg×5 days (Fig. 1A, MA vs. NMA, p = 0.0079) [25].

### Myeloablative TMZ Results in the Depletion of CD4^+^, CD8^+^ and CD4^+^Foxp3^+^ T Cells

**Figure 2:**
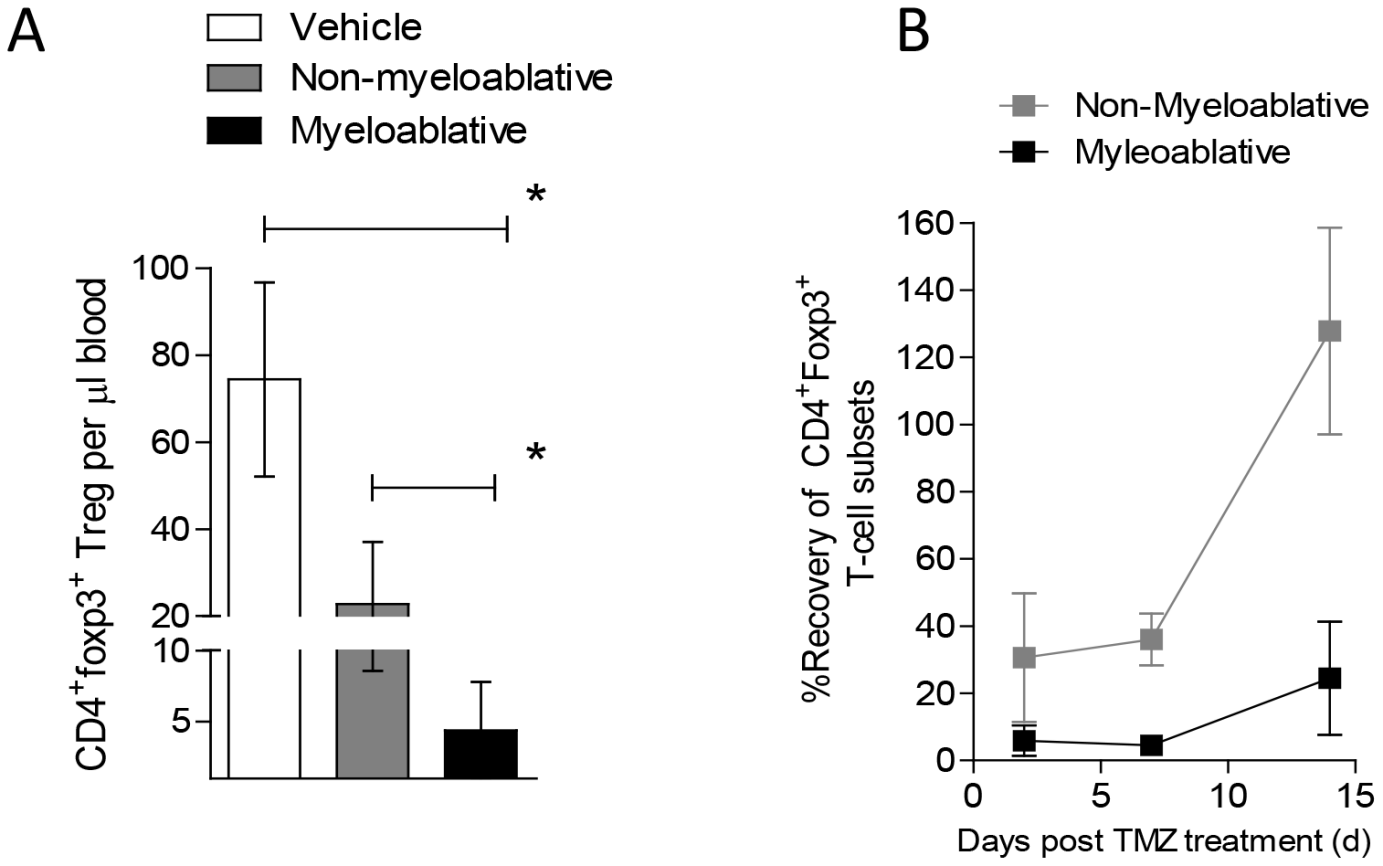
Myeloablative TMZ results in protracted depletion of CD4^+^Foxp3^+^ **T cells.** To evaluate, the impact of different TMZ regimens on CD4^+^Foxp3^+^ T_Regs_ C57BL/6 mice (n = 5) were treated with vehicle, NMA or MA TMZ regimens as stated before in Figure 1. Mice were bled retro-orbitally on days 2, 7, 14 and 28 after termination of TMZ administration and submitted to flow cytometry analysis. Immediate effects of TMZ on CD4^+^Foxp3^+^ T_Regs_ (A), on day 2 and the recovery kinetics over time are shown (B). Absolute numbers per microliter of blood were calculated using Flowcount^®^ beads from Beckman Coulter^©^. Representative experiments are shown; all experiments were performed in at least duplicate. Statistical analysis was performed using Mann-Whitney test. Statistical significance was determined at a *p value ≤0.05.

We decided to further investigate the impact of MA TMZ on subpopulations of the T-cell compartment. Flow cytometric analysis demonstrated that CD4^+^ and CD8^+^ T cells were profoundly depleted immediately after cessation of MA TMZ (Fig. 1C, for CD4^+^ T cells, MA vs. Veh, p = 0.0079; Fig. 1E, CD8^+^ T cells, MA vs. Veh, p = 0.0079) and also remained depressed at 28 days (Fig. 1D and F). The CD4^+^/CD8^+^ T-cell ratio was maintained over the period analyzed (data not shown). Concomitant analysis of CD4^+^Foxp3^+^ T_Regs_ also revealed severe depletion following MA TMZ (Fig. 2A, MA vs. Veh, p = 0.0119) and protracted recovery compared to NMA TMZ-treated mice over 2 weeks (Fig. 2B). Of note, the NMA, lymphodepletive TMZ regimen resulted in a lesser degree of depletion in CD4^+^ T cells (Fig. 1C, MA vs. NMA, p = 0.0079), CD8^+^ T cells (Fig. 1E, MA vs. NMA, p = 0.0079) and CD4^+^Foxp3^+^ T_Regs_ (Figure 2A, MA vs. NMA, p = 0.0079). These data demonstrate that conventional CD4^+^ and CD8^+^ T cells, as well as CD4^+^Foxp3^+^ T_Regs_, are sensitive to TMZ, however higher doses of TMZ were necessary to achieve sustained T_Reg_ depletion.

### Myeloablative TMZ Increases Antigen-specific CD8^+^ T-cell Expansion and Survival Following Vaccine

**Figure 3:**
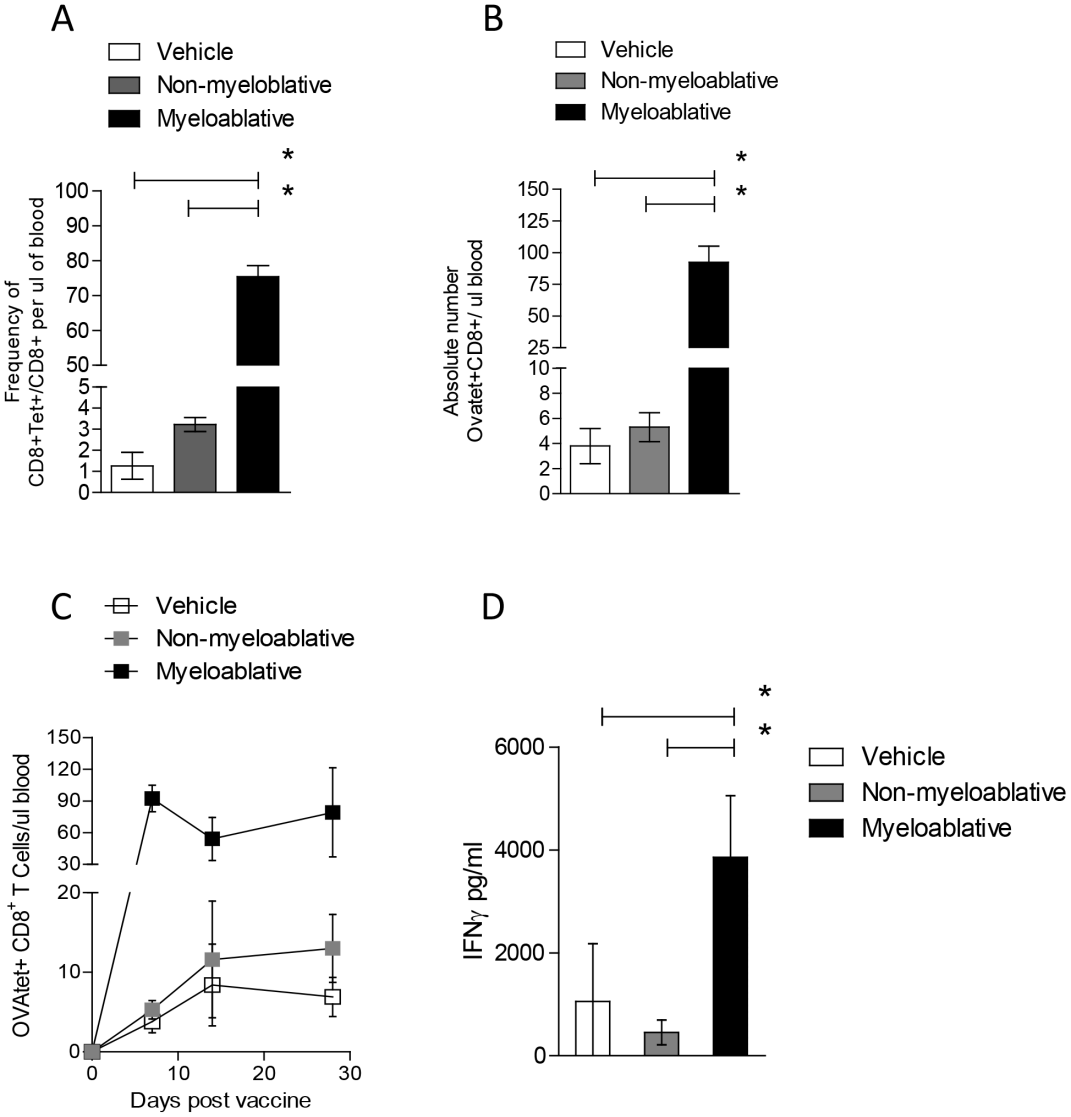
Myeloablative TMZ increases antigen-specific CD8^+^ T-cell expansion and survival following vaccine. Antigen-specific CD8^+^ T-cell responses were investigated in wild-type C57BL/6 mice (n = 5). Mice were treated with vehicle, NMA or MA TMZ intraperitoneally for 5 consecutive days. The day after TMZ treatment termination, mice received an intravenous adoptive transfer of 1×10^7^ splenocytes consisting of 1∶1 ratio of OT-I and C57BL/6 splenocytes. Mice underwent subsequent intradermal vaccination with 100 µg of SIINFEKL chicken Ovalbumin epitope, emulsified in CFA. The frequency (A) and absolute number (B) of OVA-specific T-cells were monitored in peripheral blood using an OVA-specific tetramer and anti-CD8 mAb on days 7, 14 and 28 (C). Absolute numbers per microliter of blood were calculated using Flowcount^®^ beads from Beckman Coulter^©^. To evaluate the level of T-cell function in mice receiving immunotherapy in the context of NMA TMZ regimen and MA regimen we performed CBA analysis (D). Splenocytes from mice receiving TMZ followed by OT-I transfer and vaccine, were harvested on day 7 after vaccination and cultured in the presence of 1 µM SIINFEKL chicken Ovalbumin epitope (cognate) or negative peptide control (non-cognate) for 72 hours in T-cell media alone. Supernatant was harvested and samples subjected to CBA analysis as specified by the manufacturer for murine IFNγ (D). Data presented represents the cognate responses with the subtracted non-cognate background. Representative experiments are shown; all experiments were performed in at least duplicate. Statistical analysis was performed using Mann-Whitney test. Statistical significance was determined at a *p value ≤0.05.

Based on the known immunopotentiating effects of lymphopenia as well as the sustained depletion of suppressive T_Regs_ observed in MA TMZ-treated mice, we hypothesized that the host environment following myeloablation with TMZ would provide an ideal setting for antigen-specific T-cell responses to a peptide vaccine. In MA mice, we observed an enriched frequency (over 70%) of OVA-specific T cells within the circulating CD8^+^ T-cell compartment (Fig. 3A). This frequency was significantly higher than that observed in mice receiving NMA TMZ or vehicle control (Fig. 3A, MA vs. NMA, p = 0.0079). Absolute counts of circulating antigen-specific OT-I T-cells were elevated in MA TMZ-treated mice to levels that correspond to nearly one-third of the natural CD8^+^ T-cell compartment (Fig. 3B, MA vs. NMA, p = 0.0119). We also observed that, regardless of TMZ dose, memory T cells induced in TMZ-treated mice were sustained with minimal T-cell contraction, and that this ultimately led to an overall greater magnitude of the memory cell compartment in MA TMZ-treated mice over time (Fig. 3C). These data demonstrate that MA TMZ can be used to generate very high frequencies and absolute counts of both effector and memory T cells *in vivo*.

### Myeloablative TMZ Enhances T-cell Function yet IL-2 Remains Limiting

**Figure 4:**
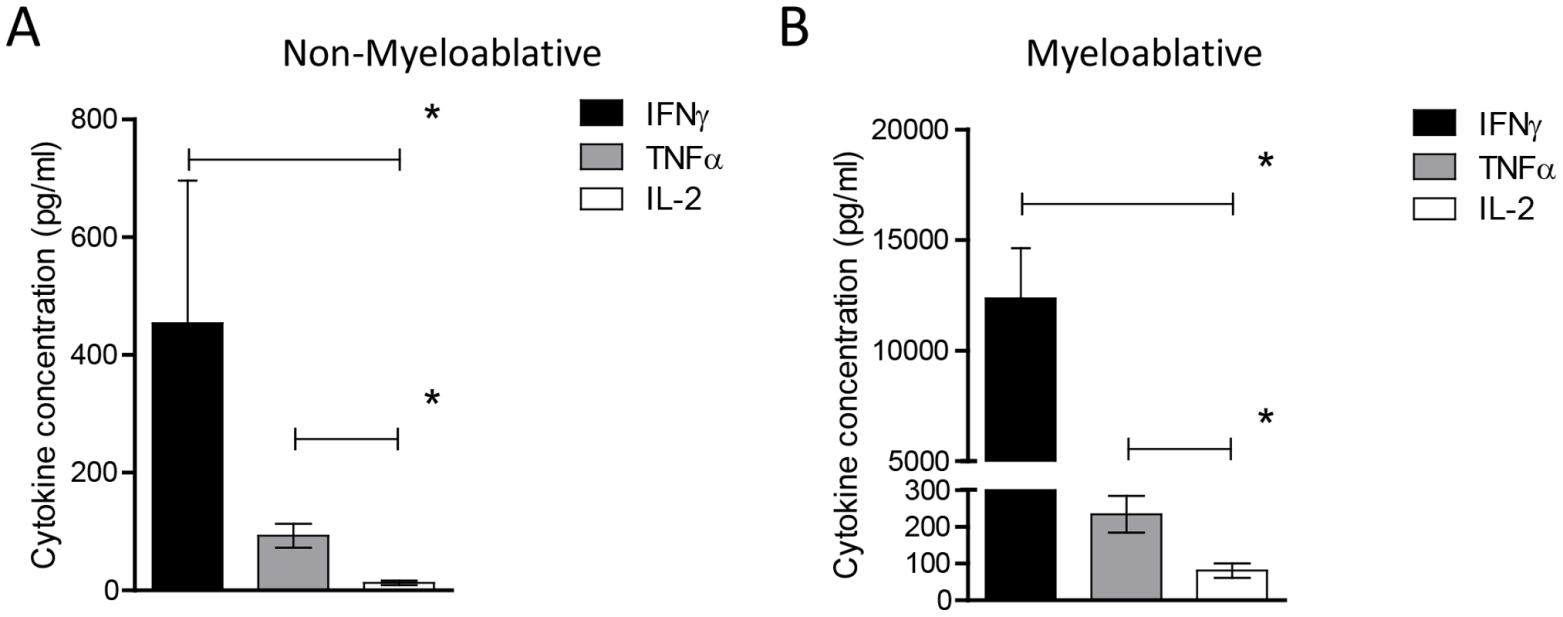
Myeloablative TMZ enhances T-cell function yet IL-2 remains limiting. To compare the levels of IL-2 secretion by OT-I T-cells relative to IFNγ and TNFα levels in the context of NMA (A) and MA (B) TMZ regimens we performed CBA analysis. Splenocytes from mice receiving TMZ followed by OT-I transfer and vaccine, were harvested on day 7 after vaccination and cultured in the presence of 1 µM SIINFEKL chicken Ovalbumin epitope (cognate) or negative peptide control (non-cognate) for 72 hours in T-cell media alone. Supernatant was harvested and samples were subjected to to CBA analysis as specified by the manufacturer for murine IFNγ, TNFα and IL-2. Representative experiments are shown; experiments were performed in at least duplicate. Statistical analysis was performed using unpaired, two-tailed student’s t-tests. Statistical significance was determined at a *p value ≤0.05.

While the presence of antigen-specific T cells is certainly a necessary component of an effective antitumor response, we decided to further assess the capacity for TMZ-induced T cells to mediate effector functionality upon antigen recognition. To do this, we performed cytometric bead array analysis on *ex vivo* stimulated lymphocytes from TMZ-treated mice. Our data demonstrate that, compared to cells isolated from NMA TMZ-treated mice, OVA-specific T cells from mice conditioned by MA TMZ displayed enhance secretion of the inflammatory cytokine IFNγ (Fig. 3D, MA vs. Veh, p = 0.0317, MA vs. NMA, p = 0.0079) and TNFα (data not shown). Interestingly, IL-2 secretion by adoptively-transferred T cells was limited in comparison to other measures of immune function in both NMA- (Figure 4A, IFNγ vs. IL-2, p = 0.0152; TNFα vs. IL-2, p = 0.0010) and MA-treated mice (Figure 4B, IFNγ vs. IL-2, p = 0.0111; TNFα vs. IL-2, p = 0.0047).

Importantly, IFNγ, TNFα and IL-2 have been cited together as a complement of cytokines that define specialized polyfunctional T cells, which in turn represent a highly active subset of effectors in the antitumor immune response [34]. Thus, while our data demonstrate that MA TMZ conditioning has the capacity to augment effector T cells with regard to expansion, function and proinflammatory cytokine secretion (*i.e.*, IFNγ and TNFα) we reasoned that the relative absence of IL-2 production from transferred cells may critically limit the capacity to achieve optimal T-cell activity.

### Myeloablative TMZ Leads to Elevated IL-2 Serum Levels, which are Required for Optimal Antigen-specific T-cell Expansion

**Figure 5:**
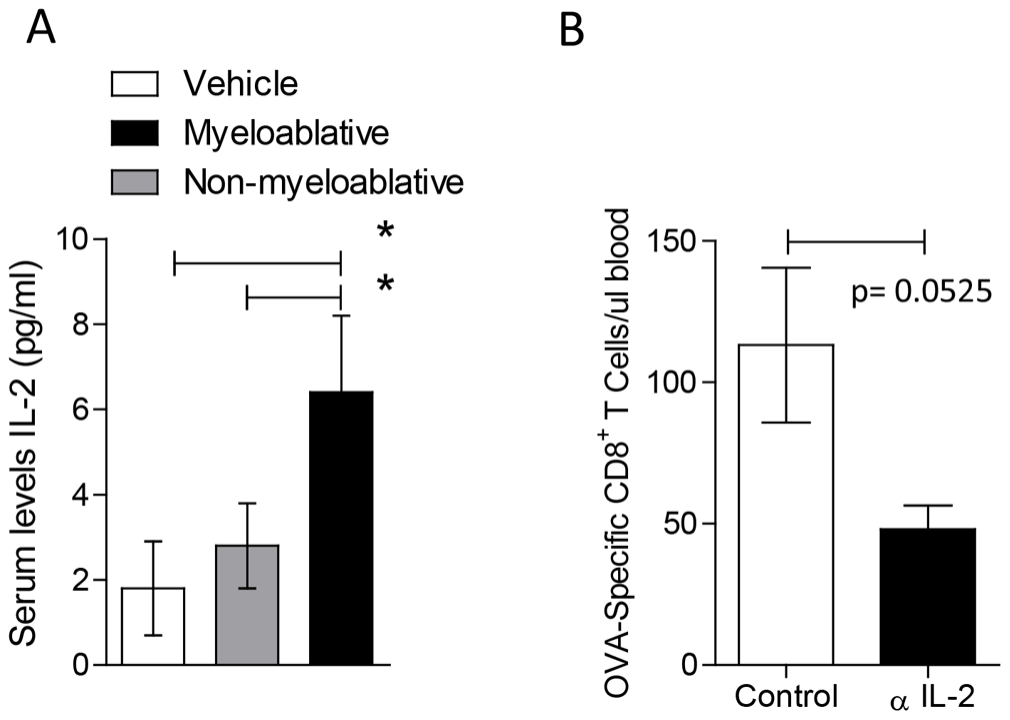
Myeloablative TMZ leads to elevated IL-2 serum levels, which are required for optimal antigen-specific T-cell expansion. The presence and requirement of endogenous IL-2 serum levels for optimal antigen-specific T-cell expansion following myeloablative TMZ was evaluated in wild-type C57BL/6 mice (n = 5 per group). To investigate the levels of endogenous IL-2, mice were treated with vehicle, NMA or MA TMZ for 5 consecutive days. Myeloablated mice received an HSC rescue on the day after cessation of TMZ treatment. (A) Serum IL-2 levels were analyzed 5 days after cessation of TMZ treatment by LINCOplex analysis. Statistical analysis was performed using Mann-Whitney test. Statistical significance was determined at a *p value ≤0.05. (B) To assess the role of IL-2 in potentiating antigen-specific T-cell responses, mice were infused with anti-IL-2 antibodies (S4B6 and JES6-1,250 µg per mouse, intraperitoneal), followed by OT-I ALT transfer and peptide vaccine in CFA. Anti-IL-2 antibodies were administered every other day for 7 doses. Mice were bled on day 7 and blood was stained with anti-CD8 and OVA tetramer w\as described before on Figure 3. Absolute numbers per µl of blood were calculated using Flowcount^®^ beads from Beckman Coulter^©^. Statistical analysis was performed using unpaired t- test. Statistical significance was determined at a *p value ≤0.05.

Previous studies have shown that, because T_Regs_ express high levels of IL-2Rα, their depletion may lead to elevated IL-2 serum bioavailability and subsequent potentiation of immune responses. Because we observed sustained T_Reg_ depletion specifically in MA TMZ-treated mice compared to NMA TMZ, we hypothesized that serum IL-2 levels would be enhanced in the setting of MA TMZ. Expectedly, we found that indeed, the MA TMZ regimen led to a sustained increase in circulating levels of IL-2 that was not observed at a lower NMA TMZ dose (Fig. 5A, MA vs. Veh, p = 0.0079; MA vs. NMA, p = 0.0079).

Based on these data, we reasoned that the post-myeloablative surge in serum IL-2 might serve to supplement the function of adoptively-transferred T cells which–although proficient in IFNγ and TNFα secretion–were otherwise singularly deficient in IL-2. To evaluate the impact of endogenous IL-2 on T-cell expansion in MA TMZ-treated mice, we performed targeted IL-2 blockade prior to ALT and vaccine. We found that IL-2 blockade in this setting led to a significant decrease in circulating antigen-specific OT-I T-cells compared to mice receiving vehicle, supporting a critical role of serum IL-2 in MA TMZ-treated mice (Fig. 5B, p = 0.05). Importantly this decrease was not significant in mice receiving IL-2 blockade in the context of NMA TMZ (data not shown). These data illustrate a novel aspect of MA TMZ that has not been previously observed using other NMA or MA regimens, namely the induction of elevated serum IL-2, which was required for optimal expansion of antigen-specific T cells upon vaccination.

### Myeloablative TMZ is Required for Efficacious Immunotherapy against Intracerebral Tumor

In current clinical practice and investigational studies, TMZ is used in four different regimens that vary in the degree of resulting lymphopenia. Because we observed that serum IL-2 was sustained specifically in MA TMZ-treated mice but not those treated with NMA TMZ, we sought to determine whether myeloablative TMZ would be superior to the previously-published NMA TMZ dose with regard to its ability to augment antitumor immunotherapy against experimental brain tumors.

**Figure 6:**
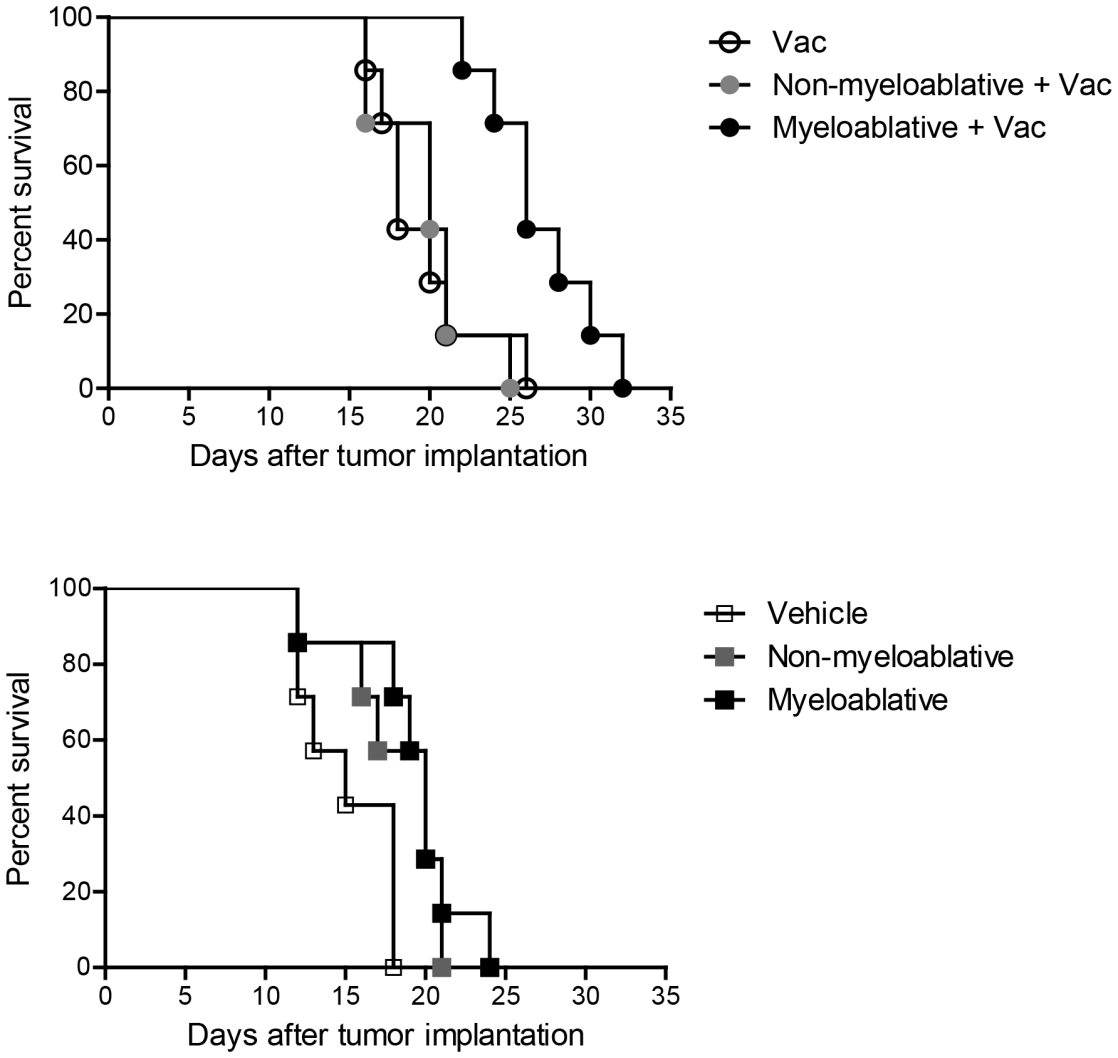
Myeloablative TMZ is required for efficacious immunotherapy against intracerebral tumor. Antitumor efficacy of a vaccination in the context of lymphopenia induced by the different TMZ regimens and the impact of these TMZ regimens as a single agent were investigated in wild-type C57BL/6 mice (n = 7) inoculated with an intracerebral injection of 5×10^3^ B16F10-OVA melanoma cells. These mice received vehicle, NMA or MA TMZ treatment followed by vaccine (A) or no vaccine (B). On day 3 after tumor implantation, dose-intensifying TMZ regimens were initiated and administered for 5 consecutive days intraperitoneally. Vaccinated mice received an intravenous adoptive transfer of 1×10^7^ splenocytes consisting of 1∶1 ratio of OT-I and C57BL/6 splenocytes on the day after termination of TMZ treatment. Mice underwent subsequent intradermal vaccination with 100 µg of SIINFEKL chicken ovalbumin epitope emulsified in CFA. (B) In order to assess the effects of TMZ alone, mice were also treated with TMZ in the absence of vaccine. Mice were monitored for morbidity end-points approved by the Duke University IACUC and sacrificed when end points were met. The experiment was performed three times. Survival analysis was performed using the Gehan-Breslow-Wilcoxon Test. Statistical significance was determined at a *p value ≤0.05.

Our data show that vaccination in mice treated with the NMA TMZ regimen failed to prolong survival in mice with intracerebral tumors, achieving similar median survival as mice receiving vaccine alone (Fig. 6A). By contrast, vaccination in the context of MA TMZ significantly improved antitumor immune efficacy in mice with established intracerebral tumors, leading to prolonged median survival (Fig. 6A, Vac vs. MA, p = 0.0031; NMA vs. MA, p = 0.0019). Importantly this effect was agnostic of any direct influence of TMZ on tumor progression since administration of MA TMZ alone did not significantly impact survival when compared to mice treated with NMA doses (Fig. 6B). Albeit this survival benefit was modest, these data support that MA TMZ is required for efficacious immunotherapy against intracerebral tumors.

## Discussion

In multiple settings, it has been shown that lymphopenia can be strategically manipulated to enhance antitumor immune responses [9]–[12]. Our data suggest that similar effects might be achieved against tumors in the brain using TMZ as a host-conditioning regimen, but only at elevated, myeloablative doses. Our group previously showed that lymphodepletive TMZ augments vaccine-induced antitumor immune responses against subcutaneous tumors in the periphery [16]; by contrast, an unexpected finding in this study is the observation that mice with intracerebral tumors did not exhibit prolonged survival at NMA doses. Our current study demonstrates that MA TMZ, but not lower lymphodepletive doses are required in order to achieve survival benefits following adoptive immunotherapy in mice with intracerebral tumors.

A number of mechanisms may be contributing to the ability of MA TMZ to effectively enhance antitumor immune responses specifically against intracerebral tumors. T_Regs_ are frequently implicated as a central cause of host immune suppression and have been shown to be elevated in the peripheral blood of patients with brain tumors. In our study, we report that MA TMZ, but not NMA TMZ successfully achieved sustained depletion of CD4^+^Foxp3^+^ T_Regs_ in the periphery. Certainly, the unique impact of MA TMZ on the circulating T_Reg_ compartment may be relevant to its improved ability to effectively potentiate immune-mediated antitumor effects in the CNS [35]. Due to the blood-brain barrier, tumors in the brain are also traditionally thought to have limited access to circulating lymphocytes, which may make them less susceptible to immune-based approaches. However, it is also known that specifically upon activation, T lymphocytes traffic frequently into the CNS to mediate effector responses there [36]. While the absence of IL-2 has been shown to antagonize T-cell activation, the surge in serum IL-2 following treatment with MA TMZ may conversely potentiate both the magnitude and activation status of the responding T cells, leading to enhanced localization and efficacy against intracerebral tumors.

Lending credence to its importance in the antitumor immune response, exogenous IL-2 is generally required for efficacious adoptive therapy. However, the elevation of endogenous IL-2 following MA TMZ may ultimately obviate the need for high-dose IL-2 administration, which is contraindicated in some patients with CNS tumors [37], [38]. Moreover, given that adoptively-transferred T cells in our study were not observed to secrete high levels of IL-2, treatment with MA TMZ provides a key factor that is absent, but otherwise necessary, in what has been described as a potent antitumor polyfunctional T-cell response; importantly, blockade of serum IL-2 specifically in MA TMZ-treated mice led to significantly decreased T-cell expansion in our model.

Despite the finding that MA TMZ was required for antitumor efficacy in our study, the median survival benefit was modest (6–9 days). One limitation of this study is that it did not account for tumor-associated suppressive cells (*e.g.*, myeloid-derived suppressive cells, macrophages, T_Regs_) that might hamper an otherwise potent antitumor immune response. Importantly, we observed that circulating T_Regs_ eventually reached normal levels after treatment with MA TMZ. We speculate that if circulating T_Regs_ are any indication of their relative presence in the intratumoral microenvironment, this could provide a possible explanation for limited efficacy in our study.

Although increased precursor frequency would in theory improve antitumor efficacy in our model, recent studies suggest a more complex association between dose upon adoptive transfer and tumor response [34]. In our model, we found that efficacy was not significantly altered as a function of doses (5×10^3^–10^6^ cells) resembling equivalents spanning both natural and supraphysiological precursor frequencies (data not shown). Thus, we believe that precursor frequencies may not play a critical role in the context of MA TMZ.

Here we have reported a viable approach for inducing lymphopenia and enhancing antitumor immunity that leverages the side effects of TMZ, a well-tolerated, clinically approved chemotherapy for multiple tumor types. Our study highlights the importance of high-dose, MA TMZ as a host-conditioning regimen for immune-based treatment of tumors in the brain, and suggests that this strategy may ultimately obviate the use of exogenous IL-2 in this setting. While standard TMZ regimens for GBM patients currently results in moderate grade II lymphopenia, MA TMZ is currently under phase I/II evaluation as a single agent for patients with high grade gliomas [6], [39]. Thus, these data contribute to the growing body of literature supporting TMZ as an alternative to conventional lymphodepletive regimens and have implications on future clinical trial design and immunotherapeutic approaches for brain tumors.
